# Influence of Micro-Organisms in the Production of Caries

**Published:** 1884-11

**Authors:** Arthur S. Underwood

**Affiliations:** Lecturer on Dental Anatomy and Physiology at the Dental Hospital of London Medical School, etc.


ARTICLE II.

### ON THE INFLUENCE OF MICRO-ORGANISMS IN THE PRODUCTION OF CARIES.

BY ARTHUR S. UNDERWOOD, M. R. C. S., AND L. D. S. ENG.

Lecturer on Dental Anatomy and Physiology at the Dental Hospital of London Medical School, etc. *

(Read before the Odontological Society of Great Britian, April 7, 1884.)

*Mr. President and Gentlemen:*

Mr. Milles and myself have so much to lay before you to-night in the form of evidence concerning the part played by micro-organisms in the production of caries, that we feel confident you will excuse us if we go straight to the matter in hand without an unnecessary waste of time in the form of preamble.

It is, however, necessary to state that we only propose to discuss the purely pathological side of the question to-night, and that only so far as the dentine alone is affected.

As we stated in 1881, we still consider that the initial stage, that is, the enamel destruction, is probably little more than a chemical solution of the tissue by an acid or acids; but we are inclined to believe that the greater part of such acids are the result of fermentation, a process which is entirely dependnet upon micro-organic life, as has been amply proved by Pasteur, Lister, and others.

As the result of the decomposition of albumenoids, numerous substances are formed in the mouth—peptones and other similar bodies, fatty acids, acetic, formic, butyric

*By the courtesy of the author, and with the special permission of the Society, we are furnished with corrected proof-sheets of the *Transactions of the Odontological Society of Great Britian*, for April, containing the above paper. The discussion that followed the reading of this paper we hope to give in our next number.—ED.

malic, lactic, &c., and in some cases sulphuretted hydrogen and ammonia are produced by the vegetation of micro-organisms.

A very simple illustration of this fact is obtained by filling two bottles with neutral saliva and bread, and rendering one aseptic, either by adding carbolic acid or boiling, or any other process of the kind. The micro-organic growth which will take place in the impure one will produce a large quantity of acid; the pure one will remain neutral. The most constant source of an acid reaction in the mouth is undoubtedly micro-organic fermentation.

This is not, however, the main subject of to-day's paper, and therefore we shall content ourselves with this passing allusion to it.

We cannot, however, refrain from expressing our gratification at the careful attention this subject has received from the profession, both here, in America, and on the Continent. Professor Mayr has worked out the chemical aspect of the question with great care and exactness; Mr. Tomes has done much in the direction of the effects of organisms in soft tissues of the teeth; Mr. Spence Bate has given us many valuable suggestions in his able and exhaustive summary of dental science at Plymouth last year; Mr. Sewell has thrown our valuable conjectures upon the remote etiology of caries; and Dr. Miller, of Berlin, has done an immense amount of microscopical work, contributing a large array of evidence.

We have not been able untill now to present our new views in full, because many of our experiments required a ong time to verify and confirm; some afforded perplexing and almost contradictory results, requiring a long time to clear up and involving much repetition, and we may add, in explanation of the apparent delay, that what takes but little time to write or say often requires months and years of disappointment and patience to work out.

In the autumn of 1881 we were only justified in speaking with any amount of positiveness upon one point, namely, the actual presence of micro-organisms in carious dentine. This one fact, and the evidence in support of it, formed the body of the paper we ventured to present to the International Congress. Micro-organisms were found in every fragment of carious dentine of which we cut sections, and we had cut immense numbers; the leptothrix of Lebel and Rottenstein was, we found, a superficial growth.

Another fact of scarcely less importance, but one supported by far less evidence, was that the agencies to whose action caries had up till then been attributed by a large majority of scientific opinions, namely, certain acids, were unable to produce any such changes upon teeth exposed to their action alone, although the enamel protection was removed, and the acids employed were those present in the mouth. We had read many allusions to caries produced in teeth out of the mouth by artificial means; we employed the means indicated (whenever they were indicated, which was rarely,) with the addition that we did so under absolutely aseptic conditions . we repeated the experiments, and the results of (in some cases) years of exposure was that no change that we could detect took place. Here was a second, but a negative fact.

The third fact obviously required was a positive one; it remained to inoculate dental micro-organisms into the flnaks add observe the result. We did so, and the result was change in the exposed dentine, but so slight that we could not succeed in cutting a section; still, at the time of our paper of 1881, we firmly believed this change, when allowed to become more intense, would prove to be caries. Of the third fact we felt bound, however, to speak very cautiously, and for the last two and a half years we have employed every means in our power to clear it up—with what result we shall endeavor to make plain to you to-night

We are anxious that you should keep clearly before you four main issues, upon which all the theory which we suggested to you in 1881 depends.

1. That certain forms of micro-organisms, namely, micrococci, rod-shaped oval, bacteria, and short bacilli are invariably present in carious dentine.

2. That these micro-organisms extend into the tissue as far as does the caries.

3. That no agents can be made to produce a change resembling caries in the absence of such micro-organisms, i. e., under asceptic conditions.

4. That under septic conditions a change can be induced which, although we are not prepared to call caries, does in some particulars resemble it; teeth in which this change has been produced we shall submit to you to-night, and also, suggest to you why we hesitate to call it caries.

These four points once established upon reasonable evidence, we shall ask you to consider whether we are not justified in deducing from them the conclusion that micro organisms play an important part in the productiou of the disease as we see it and are called upon to treat it, and that their active presence is an absolute essential to its production.

We may add, incidentally, that we never suggested that no other agency assisted in the process, although a recent writer attributes some such absurdity to the advocates of the germ theory. We do not consider ourselves justified in wasting your time over such a digression. The first point to discuss, then, is the constant presence of micro-organisms in carious dentine. Upon this point the evidence submitted to the profession by us in 1881 was very strong. We were then able to state that, since we had succeeded in properly cutting and staining sections, we had always discovered mibro-organisms. The appearance was not caused by softening agents, because the teeth were cut fresh and immediately after extraction. The number of sections cut by us between 1879 and 1881 was so great that we were convinced that the phenomenon was constant.

Since then, Mr. Charles Tomes has, we believe, made some investigations which have led him to concur in this constant presence.

Dr. Miller, of Berlin, has cut a very large number of sections of carious dentine, with a result which is completely confirmatory of our assertion that micro-organisms are invariably present. In fact, this point has been confirmed by every careful observer, and may be considered to be completely established.

The second point is very much more difficult to decide—the more so because statements have been made upon this point which tend very much to confuse the mind of the reader. It has been stated by Dr. Miller, who has written a very large number of papers upon this and kindred subjects during the last two years, that there is a zone of softened dentine of considerable width, not affected with micro-organisms, separating the normal dentine from that which is so infected; and further, that the outline of this area of softened dentine does not correspond with the outline of the area infected with organisms. We have attempted to verify this observation, but we have arrived at the conclusion that if there be any such zone at all we cannot believe it to be of any considerable depth, as we have not succeeded in finding any softened tissue that did not contain micro-organisms. Having regard, however, for Dr. Miller's reputation as a careful and conscientious explorer, we do not desire to attach too much weight to negative evidence; later on we shall suggest some weighty positive evidence on this point. With regard to the outline, we have invariably found the outline of the changed tissue to corrrepond with that occupied micro-organisms, and we feel less hesitation in doubting this latter statement of Dr. Miller because he claims to be able to compare these outlines with the naked eye, unaided by any instrument. We think that this is too rough a test for objects whose greatest measurement does not exceed the one-one-thousandth of an inch.

There is no doubt that it is easy to establish a line of demarcation with the aid of a sharp instrument between tissue that you can easily prick and that which is quite hard; this line no doubt corresponds very nearly to the edge of the disease. We have also come to the conclusion that corresponds very nearly to the edge of the micro-organic invasion; that is, we have cut sections as near as we could to the healthy tissue and found them infected. We do not, however, attach so much weight to this fact as to be following experiment, which we think must be absolutely conclusive.

Before we can explain its full weight we must enter into a few details of the method we employ to grow and cultivate special germs. We used to make a meat infusion, boil it, introduce it with antiseptic precautions into asceptic test tubes, and seal the tube with wool. If the fluid remained clear we knew it was pure; if it become turbid within a day or two we knew our precautions had not been complete, and some germs had been introduced accidentally, and the tube was of course useless. If we wished to grow a special germ we rubbed a purified probe against the infected spot, and introduced it into the fluid, replaced the wool cap, and the ensuing turpidity denoted the growth of the germ. The objection to this plan was that the turpidity spread through the entire fluid, and if we had, despite our precautions, introduced any other organisms besides those we specially intended to introduce, they all become inextricably mixed in the ensuing putrefaction, To obviate this, we adopted Koch's plan of adding gelatine, and instead of a pure fluid, making a pure jelly: when this inoculated in a particular spot the growth was confined to that spot, and if anything else dropped on the surface of the jelly it grew there quite separate. Under these circumstances we noticed that different forms of germ grew in quite different ways—some, for instance, forming a white ball, others a mass of spiculæ, others wavy lines; these peculiarities of growth could be easily reproduced in other flasks by a second inoculation, until we found it quite easy to separate each variety and grow it by itself without any extraneous growth to confuse the result. We have brought down some flasks, with growth going on, to show you to-night Well, we proceeded to inoculate flasks with those deepest portions of softened tissue which formed the most outlying layer of changed dentine, and we found that from these portions a growth of organisms proceeded, proving beyond doubt that organisms were contained in the pieces inoculated. This is not a doubtful experiment; it absolutely demonstrates that the deepest portions of the softened tissue contained organisms, and if there exist any zone of alterations beyond them it is so microscopically narrow that we have not been able to detach a fragment of it.

We do not, however, deny that there is a great probability that the advance guard of micro-organisms may be surrounded, and, *to a microscopical extent only*, preceded by a softening, the result of the action of those acids which they themselves produce. We shall be able to show that they do generate acids capable of softening dentine, and therefore we should expect to find this effect slightly in advance of their presence; but this is hypothesis merely. We would further add that the transition from very carious tissue to that which seems quite healthy to the eye and the touch is, in the greater number of cases, more sudden than is generally supposed, and allows but little space for this non-infected zone. To convince oneself of this, it is only necessary to split a number of carious teeth and prick the surfaces. One more fact should be borne in mind in this connection, namely, that healthy dentine softened by a weak acid does not look different under the microscope from healthy dentine cut fresh, and it is therefore impossible to detect the softening microscopically; the tubes are not larger, nor are they irregular, as is the case in carious dentine—in fact, the existence of such a softened and uninfected zone separating the infected from the non-infected dentine is s matter so difficult to demonstrate that we do not think much value is to be attached to it.

We now come to the third point, viz.: that it is not possible to produce caries, or anything resembling it, by subjecting teeth to the action of acid fluids, in flasks out of the mouth, provided the flasks be kept in a strictly asceptic condition. We do not think this point has ever been disputed, and, we feel in position to state that the evidence upon which it rests is incontrovertible; but, before discussing it, and the point that follows it, we think it will not be a waste of time to explain what we mean by a change resembling caries. We mean a change by which the tissue affected is made distinguishable from that which is normal to the naked eye and touch or under the microscope. To the naked eye it must become discolored—we only mean changed in the color, and not necessarily brown; the surface loses its polish, or, if moist, the affected part must be separable from the healthy with an instrument; to the touch it must become softer; under the microscope the channels must become dilated at the expense of the matrix. In 1881 we exhibited several flasks in which infusions of meat and saliva containing malic, butyric and other acids had been allowed to act upon exposed dentine for periods varying from one to three years; the teeth were liable to caries because they had suffered already from it; healthy portions were denuded of enamel and partially protected by wax, a certain part being left exposed with no perceptible result whatever. Of course a strong acid, or a large amount of acid, would decalcify the tooth, but this does not resemble carious change.

These experiments were sufficiently numerous and conclusive to warrant us in passing on to the fourth point of this investigation, and this will detain us longer than any of the others, as it is in this direction that we have experienced the greatest difficulties and the most perplexing results.

We were so fully alive to the overwhelming difficulties attending any attempt to reproduce out of the mouth the complicated conditions existing therein, that we should have felt anything but sanguine but for a curious accident that encouraged us to proceed in this direction. We will therefore commence by telling you the particulars of this accident.

At the Congress in August, 1881, we exhibited, among other flasks, a series intended to illustrate the fact that no change resembling caries could be induced upon the surfaces of teeth hnder aseptic conditions. The flasks all contained an infusion of meat and saliva with malic and butyric acid and teeth susceptible of caries, the surfaces ground down to expose the dentine, part of the exposed surface being protected with wax, so that any loss of substance could be at once detected in the exposed part. Now these flasks, which were labeled i, iii, iv, v, vi, had remained perfectly pure from the time they were put up, June, 1880, till the Congress, August, 1881, that is, fourteen months, and no visible change had taken place in the exposed surfaces. In taking them home in a bag, flasks, i, iv, and v fell down, allowing the fluid to come in contact with the cap covering the flasks; iii and vi remained upright.

In the course of a few days, flasks i, iv and v, which had tumbled down, became turbid; iii and vi remained, of course, pure. By the 5th of December, 1881, iii and vi were in all appearances, as before, unaltered; but the teeth in i, iv, and v were already discolored brown; moreover, the discolored parts were on the surfaces slightly softened, and although the change was so shallow that we could not obtain a good thin section of it, we could see that we could not obtain a good thin section of it, we could see that the tubes were enlarged and contained a material that stained readily, which, owing to the thickness of the section, we could not, unfortunately, identify. Now, whatever this change was, it was manifestly dependent upon the introduction of organisms; nothing else was introduced. Fourteen months' immersion in ths fluid, when asceptic, failed to change the teeth at all; four months in the same fluid, plus germs, produced a change; the same additional four months, in the sister flasks, produced no change at all This seemed an important addition to the facts in our possession at the Congress. The next stage was less satisfactory; the change did not seem to progress; do what we would, we could not produce a considerable change. We conceived that this might arise from the fact that several important conditions favorable to the activity of the germs in the moutn were absent in the flask. First, the temperature of the body; second, opportunities for lodgment; third, constant renewal; fourth. unlimited and varied pabulum; we threfore contrived a very elaborate experiment in which we proposed to assemble all these conditions. and as many more as we could think of. We erected a large incubator, which we kept continually at the temperature of the mouth; it was divided into an upper and a lower story, which communicated by an aperture. In the upper story was a small bath with a tiny tap contrived to drip drop by drop through the aperture into a second bath in the chamber below; this again dripped into a receiver, the fluid being thus kept in constant circulation through the mindle bath. In this middle bath were placed some teeth set side by side as in nature, the roots being implanted in a block of hippopotamus ivory, and weak points created in the interstitial parts with the burring engine, the size of the burr being kept to test any enlargement of the holes, and some other implanted in wax. The upper bath was then filled with saliva, milk, bread, decaying teeth, meat, etc.; while some fragments of the latter were inserted about the teeth in the middle bath. The lowest receptacle was periodically emptied, while the top one was periodically fed. Without troubling you with more details, we thus contrived to assemble the proper temperature, the pabulum movement, and the normal disposition of the teeth. Now six months of this highly unsavory experiment produced scarcely any perceptible change, except that all the tissues became black, the hole drilled, in the blocks and the teeth were the same size, and their edges were not a bit softened. The putridity of the baths was, however, so offensive, that it with some relief that we decided to abandon this particular experiment. We saw that some great essential condition was absent, and though a longer period might doubtless have produced slight changes, my health and appetite suffered from the constant exposure to all these putrid smells, and we agreed to stop the incubator ; but this experiment, though apparently a failure, led us to what we conceive to be the true solution of the difficulty. So far we decided only that some essential factor had been omitted.

[TO BE CONTINUED.]

